# The Impact of High Urban Temperatures on Pesticide Residues Accumulation in Vegetables Grown in the Greater Accra Metropolitan Area of Ghana

**DOI:** 10.3390/jox15040103

**Published:** 2025-07-02

**Authors:** Joyce Kumah, Eric Kofi Doe, Benedicta Yayra Fosu-Mensah, Benjamin Denkyira Ofori, Millicent A. S. Kwawu, Ebenezer Boahen, Doreen Larkailey Lartey, Sampson D. D. P. Dordaa, Christopher Gordon

**Affiliations:** 1 Institute for Environment and Sanitation Studies (IESS), College of Basic and Applied Sciences, University of Ghana, Legon, Accra P.O. Box LG 209, Ghana; joyheflide@gmail.com (J.K.); ericdoe1st@gmail.com (E.K.D.); bdofori@ug.edu.gh (B.D.O.); ebenboahen1045@gmail.com (E.B.); dlartey222@gmail.com (D.L.L.); dordaa41@gmail.com (S.D.D.P.D.); cgordon@ug.edu.gh (C.G.); 2 Department of Geography and Earth Sciences, School of Natural and Environmental Sciences, University of Environment and Sustainable Development (UESD), PMB, Somanya, E/R, Ghana

**Keywords:** food system, ecotoxicology, human health risk, food safety, consumers, accumulation

## Abstract

This study investigates the effect of high urban land temperatures on pesticide residue (PR) accumulation in cabbage and lettuce and on public health in the Greater Accra Metropolitan Area (GAMA) in Ghana. A comparative toxicological analysis regarding the food system was conducted with 66 farmers across three land surface temperatures: low (Atomic, *n* = 22), moderate (Ashaiman, *n* = 22), and high (Korle-Bu, *n* = 22). Pesticide residue concentrations were assessed using an ANOVA to examine spatial variations across sites. The results indicate a strong correlation between high land surface temperatures and pesticide residue accumulation, with lettuce recording significantly (*p* < 0.05) higher PR levels than cabbage. Several pesticides, including carbendazim (CBZ), Imidacloprid (IMI), Thiamethoxam (TMX), and Chlorpyrifos (CHL), exceeded the maximum residue limits (MRLs) set by the World Health Organization (WHO) and the European Union (EU) at moderate and high-temperature sites. carbendazim was the dominant pesticide detected, with a concentration of 19.0 mg/kg in lettuce, which far exceeded its maximum residue limit (MRL) of 0.10 mg/kg across all study sites. Statistical analyses (PERMANOVA) confirmed that land surface temperatures and pesticide types significantly influenced the PR concentrations. Public health risk assessments indicate that children are more vulnerable to pesticide exposure than adults. The toxicity hazard quotient (THQ) for organophosphate pesticides, particularly CHL and Dimethoate (DMT), exceeded safe thresholds at moderate and high-temperature sites.

## 1. Introduction

Urban vegetable farming plays a crucial role in ensuring food security and nutrition for city dwellers, particularly through the cultivation of cabbage (*Brassica oleracea*) and lettuce (*Lactuca sativa*). These leafy vegetables are rich in essential vitamins and minerals, making them a staple in urban diets [1,2]. However, their production often relies on agrochemicals to control pests and enhance yields, raising concerns about pesticide residue accumulation in food crops [3,4]. The presence of residues from neonicotinoids, carbendazim, and organophosphates in vegetables has been widely documented, with prolonged exposure posing significant health risks, including neurotoxicity, endocrine disruption, and reproductive disorders [5,6].

The risks associated with pesticide residues are further exacerbated by urbanization and climate change. Rising temperatures, increased demand for vegetables, and water stress contribute to the persistence and accumulation of pesticides in urban crops [7,8]. Research suggests that higher temperatures slow the photodegradation of certain pesticides, such as neonicotinoids, on vegetable surfaces, prolonging their presence and increasing potential exposure [9]. The urban heat island (UHI) effect, driven by the replacement of natural green spaces with concrete and asphalt, results in significantly elevated temperatures in city centers compared to surrounding peri-urban areas [10,11]. These warming trends create favorable conditions for the proliferation of pests, such as aphids and caterpillars, leading farmers to intensify pesticide use as a countermeasure [12,13]. Excessive pesticide application and the use of contaminated irrigation water further increase the risk of residue accumulation exceeding maximum residue limits (MRLs), posing severe health risks to both humans and the environment [14,15].

Several studies have reported the presence of pesticide residues in fresh vegetables, particularly neonicotinoids, carbendazim, and organophosphates, with concentrations varying depending on environmental conditions and farming practices [9,16,17]. Chronic exposure to these residues has been linked to adverse health effects, including nervous system dysfunction, immune suppression, and an increased risk of cancer [18,19]. To address these concerns, researchers have emphasized the need for sustainable agricultural practices, including integrated pest management, bioremediation, and strict enforcement of food safety regulations [20,21,22].

Despite ongoing research on climate change and urbanization, the direct impact of rising urban temperatures on pesticide residue bioaccumulation remains insufficiently explored [23,24]. This knowledge gap hinders the implementation of effective climate-smart agricultural policies and risk mitigation strategies. Understanding how urban heat affects pesticide persistence in vegetables is crucial for enhancing monitoring systems and ensuring food safety in rapidly urbanizing regions.

This study investigates the effect of high urban surface temperatures on pesticide residue accumulation in cabbage and lettuce grown in the Greater Accra Metropolitan Area (GAMA) of Ghana. The research hypothesis is that higher urban surface temperatures in the Greater Accra Metropolitan Area (GAMA) will increase pesticide residues in cabbage and lettuce. This research aims to provide insights into the relationship between urban environmental stressors and pesticide contamination by analyzing residue concentrations under varying temperature conditions. The findings will inform policy recommendations on pesticide regulation, sustainable urban agriculture, and public health interventions to mitigate food safety risks.

## 2. Materials and Methods

### 2.1. Study Area

**Figure 1:**
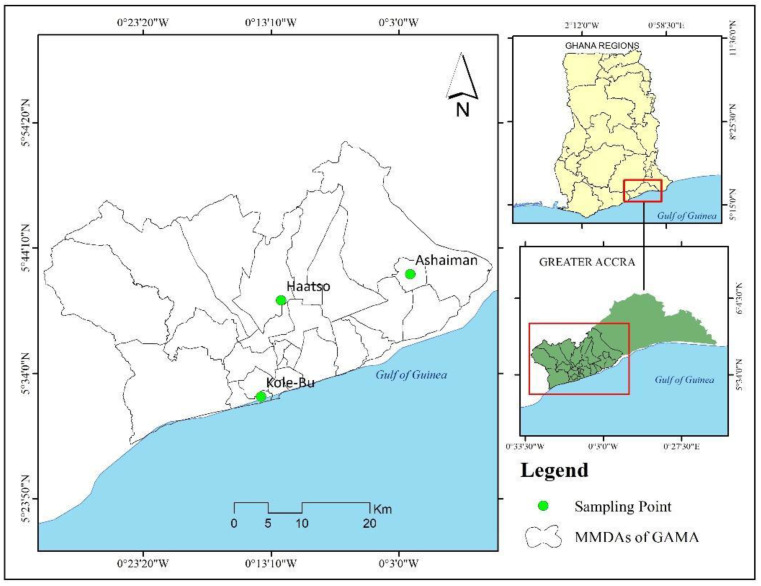
Map of study area.

This study was conducted in three urban vegetable farming sites in the Greater Accra Metropolitan Area in the Greater Accra Region of Ghana, namely “Atomic” (Haatso), Ashaiman, and Korle-Bu (Figure 1). These sites represent low, moderate, and high land surface temperatures, respectively, as characterized by Gyimah et al. [10]. Located within the Ga-East, Ashaiman, and Ablekuma-South Municipal Districts, these areas are part of Ghana’s rapidly urbanizing metropolis. GAMA covers 1507.5 km², with a population exceeding 5 million in 2021 [25]. Urban expansion has increased land surface temperatures (LSTs), affecting vegetable farming and human health in GAMA [10,26]. The study sites are characterized by coastal savannah grasslands with clay and sandy soils, where smallholder farmers cultivate crops under varied climatic conditions [27,28].

### 2.2. Study Design, Sample Size, and Sampling Procedure

This study was designed as a food system toxicological analysis involving a cross-sectional survey of 66 vegetable farmers, consisting of 33 cabbage farmers and 33 lettuce growers across three land surface temperature zones (Sites). The farmers were evenly distributed across the three farm sites, representing low (*n* = 22), moderate (*n* = 22), and high (*n* = 22) land surface temperatures with 11 farmers per crop type (Table 1). This equal sample distribution facilitated a comparative analysis of the results. A two-stage stratified random sampling approach was employed. In the first stage, cabbage and lettuce farmers were identified. In the second stage, a simple random sampling method was used to select participants from a membership list of their respective farmers’ association, which served as the sampling frame. Vegetable samples were collected from each farm for chemical analysis.

### 2.3. Sample Collection

Vegetable samples were taken on site [6]. A total of 66 samples (lettuce: *n* = 33; cabbage: *n* = 33) were carefully harvested using sterilized scissors, placed in well-labeled, sealed plastic zip-lock bags, and transported to the Ghana Standards Authority Pesticide Laboratory in Accra for analysis. Pesticide residue analysis was conducted using a multivariate approach, with pesticide residue (PR) serving as the response variable. The PR variable comprised two major pesticide groups: neonicotinoid/carbendazim and organophosphate. The neonicotinoid/carbendazim group included Acetamiprid (ACT), Clothianidin (CTD), Thiamethoxam (TMX), Imidacloprid (IMI), and carbendazim (CBZ). The organophosphate group consisted of Acephate (ACP), Azinphos (AZL), Chlorfenvinphos (CHF), Dimethoate (DMT), Monocrotophos (MON), Prochloraz (PRC), and Chlorpyrifos (CHL).

### 2.4. Sample Preparation of Pesticide Residue

Vegetable samples were extracted and purified using the QuEChERS, Newark, Delaware, USA, (Quick, Easy, Cheap, Effective, Rugged, and Safe) method [29,30,31]. The cabbage and lettuce samples were chopped into small pieces and blended separately. To prevent alterations in pesticide residue levels, the vegetable samples were not washed before processing. A 15g portion of each homogenized sample was accurately weighed and placed into a 50 mL Teflon tube, and 10 mL of 1% acetonitrile was added. The tube was sealed and vigorously shaken by hand for 2 minutes to ensure thorough mixing and efficient extraction of pesticide residues from the vegetable. An extraction salt mixture consisting of 4 g anhydrous magnesium sulphate (MgSO_4_), 1 g sodium chloride (NaCl), 1 g trisodium citrate, and 0.5 g disodium citrate was added [32]. The tube was vortexed for 2 min and centrifuged at 350× *g* for 5 min. A 6 mL aliquot of the supernatant was transferred to a 10 mL Teflon tube containing 200 mg primary secondary amine (PSA) and 150 mg anhydrous MgSO_4_ for purification (clean-up) to remove matrix interferences. The tubes were vortexed for 1 minute and centrifuged at 350× *g* for 10 min [31,32]. Finally, a 1.5 mL aliquot of the cleaned extract was transferred to GC vials and injected into a Liquid Chromatography–Tandem Mass Spectrometry (LC-MS/MS) system for pesticide analysis [32,33].

### 2.5. Pesticide Detection Using Liquid Chromatography–Tandem Mass Spectrometry (LC-MS/MS)

The vegetable samples were analyzed in triplicate for neonicotinoid/carbendazim and organophosphate pesticide residue levels (mg/kg). The samples were analyzed using high-pressure liquid chromatography coupled with mass spectrometry. A controlled flow of the mobile phase, a mixture of water and acetonitrile, carried the samples through a chromatographic column, where pesticide compounds were separated based on their interactions with the stationary phase [34,35]. After separation, the compounds were ionized via electrospray ionization (ESI) to generate charged ions, which were then fragmented and detected by the mass spectrometer.

### 2.6. Quality Control and Assurance

Strict quality control and quality assurance protocols were followed throughout the analytical process to guarantee the accuracy and reliability of the analytical results. All glassware used for sample extraction and cleanup underwent a meticulous cleaning procedure: initial washing with detergent and tap water, followed by rinsing with distilled water, thorough rinsing with analytical-grade acetone, and drying overnight in an oven at 150 °C. Once cooled, the glassware was stored in dust-free cabinets to prevent contamination.

The integrity of pesticide residue analysis was maintained through several validation steps. These included using solvent blanks and procedural matrix blanks and the analysis of samples in triplicate [36]. All reagents involved in the analysis were subjected to the same extraction protocols as the samples. Additionally, solvents were pre-screened during the analytical runtime to detect and eliminate potential interfering substances

### 2.7. Human Health Index Computation

To examine the extent of non-carcinogenic health risks of pesticide residue to consumers, a widely accepted human hazard index [30,37,38] was calculated based on an estimated daily intake (EDI) and target hazard quotient (THQ). The estimated daily intake (mg/kg/day) refers to the quantity of a particular pesticide residue that an individual ingests daily through various exposure pathways, such as consumption or inhalation, as shown in Equation (1).

(1) $$EDI=C\times EF\times ED\times \frac{1}{BW}\times \frac{1}{AT}\times IR$$

(2) $$EDI=\frac{C\times IR}{BW}$$

where *C* stands for the concentration of pesticide residue in vegetables, *EF* is exposure frequency (days/year = 365), and *ED* is exposure duration (years = 30). *AT* is the averaging time (days) (*ED* × 365), while *IR* refers to the daily intake or ingestion rate (0.345 kg/day), with *BW* representing the body weight of adults (70 kg) and children (15 kg) [30,37,38].

The THQ determined the risk associated with individual contaminants expressed as the *EDI* over the reference dose (*RfD*), which is the maximum daily exposure without adverse effects [30,38].

(3) $$THQ=\frac{EDI}{RfD}$$

The sum of the individual THQs gave the overall health risk index (HI). An HI > 1 denotes a potential adverse health effect from cumulative exposure [39,40]. The RfD values for neonicotinoid/carbendazim were as follows: ACT = 0.025, CTD = 0.009, TMX = 0.008, IMI = 0.06, and CBZ = 0.01. The RfD values for organophosphates were as follows: ACP = 0.02, AZL = 0.005, CHF = 0.003, DMT = 0.001, MON = 0.0006, PRC = 0.01, and CHL = 0.0003. Table 2 presents descriptions of pesticide residues and their human health effects.

(4) $$HI=\sum {THQ}_{i}$$

A THQ of less than or equal to one implies a safe risk or low risk of adverse health effects, while 1 < THQ ≤ 10 implies a moderate risk of adverse health effects, and there is a high risk when the THQ > 10. The same applies to HI1 < 1, 1 < HI ≤ 10, and HI > 10. The cumulative probability of an individual developing cancer over a lifetime due to exposure to possible carcinogen-causing pesticide residues was estimated using the Cancer Risk Pathway (CRP) model, as expressed in Equation (5).

CRP = EDI × CSF(5)

where CSF represents the cancer slope factor, which defines the risk of cancer per unit (mg/kg/day) exposure [52].

### 2.8. Statistical Analysis

Descriptive statistics, including percentages, means, and standard deviations, were used to analyze the data. The concentrations of individual pesticide residues were compared against the maximum residue level (MRL) thresholds set by the World Health Organization (WHO) and the European Union (EU). A composite pesticide residue variable was created by summing the individual concentrations to form a multiple-response variable.

Before statistical testing, the multiple-response variable’s normality and homogeneity of variance were assessed. Based on the results, non-parametric methods, including the Kruskal–Wallis test and Permutational Multivariate Analysis of Variance (PERMANOVA), were applied. The PERMANOVA, as recommended by Arbizu [53], is particularly suited for analyzing variance when multiple response variables are involved.

## 3. Results

### 3.1. Concentrations of Pesticide Residue in Vegetables Based on Land Surface Temperatures

**Figure 2:**
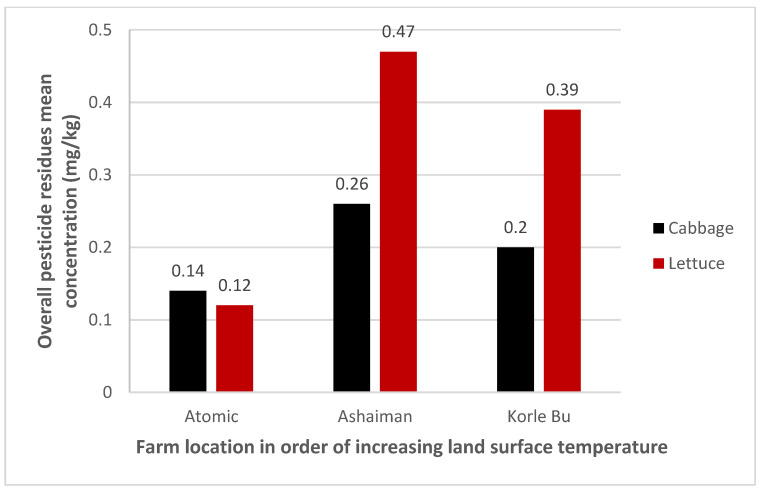
The mean concentrations of composite pesticide residues by site and vegetable.

Figure 2 presents the mean pesticide residue (PR) concentration as a multivariate response variable. The results show an increasing trend in PR concentration with rising land surface temperature. Lettuce samples recorded higher mean pesticide residue concentrations in both the moderate (0.47±) and high-temperature (0.39±) zones compared to the cabbage samples, which had lower mean concentrations (0.26± and 0.20±) in the same respective zones.

**Figure 3:**
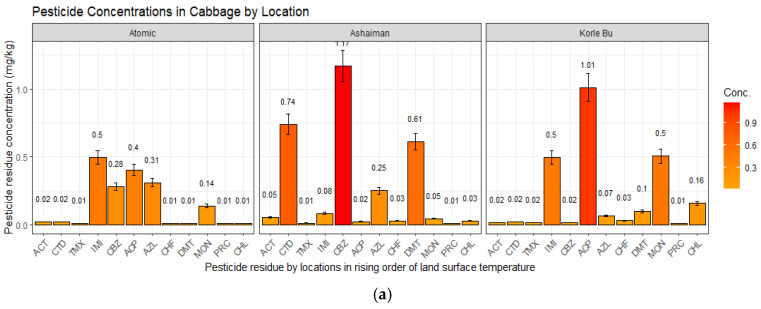

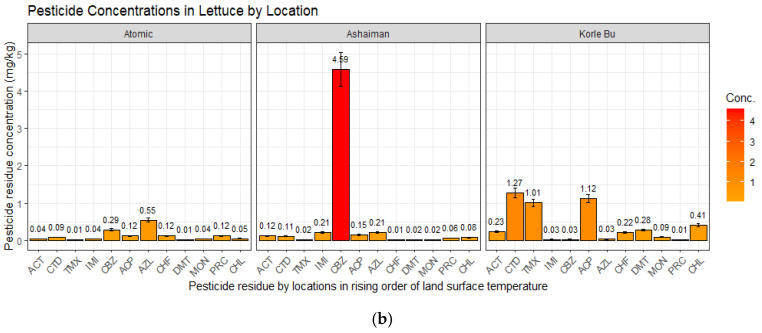
(**a**) Pesticide residue concentrations in cabbage by location in rising order of land surface temperature. (**b**) Pesticide residue concentrations in lettuce by location in rising order of land surface temperature.

Figure 3a,b present the individual concentrations of elements in the multivariate pesticide residue in cabbage and lettuce. Individually, CTD, IMI, TMZ, and CBZ pose the highest health risks to consumers of cabbage and lettuce. At the lowest temperature gradient (Atomic farm site), the concentrations of CTD (0.09 ± 0.09) in lettuce, IMI (0.50 ± 1.04) in cabbage, and CBZ (0.28 ± 0.29±) in both vegetables exceeded the WHO/EU’s maximum residue levels. At the moderate temperature gradient (Ashaiman farm site), the IMI (cabbage = 0.08 ± 0.14, lettuce = 0.21 ± 0.63), CTD (cabbage = 0.74 ± 1.09), and CBZ (cabbage =1.17 ± 2.87, lettuce = 4.59 ± 7.53) residue levels exceeded the MRLs. At the highest temperature gradient (Korle Bu farm site), the exceedance of the MLRs was most prevalent for TMX (1.01 ± 1.69) and CTD (1.27 ± 3.77) in lettuce.

**Figure 4:**
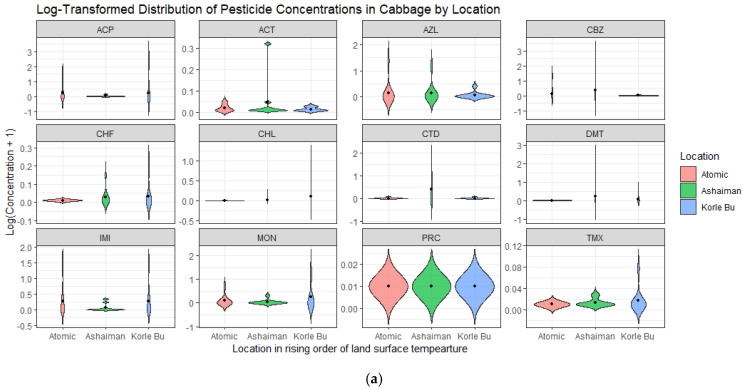

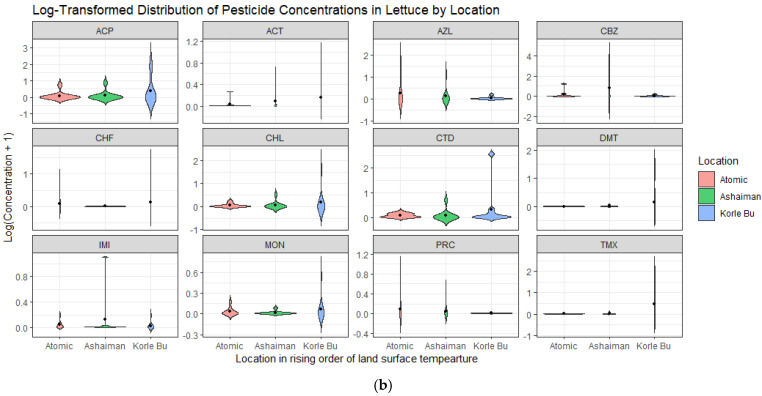
(**a**) Violin plots of the log-transformed distribution of pesticide residue in cabbage by location. (**b**) Violin plots of the log-transformed distribution of pesticide residue in lettuce by location.

Most of the measured individual pesticide residue levels clustered around the mean (Figure 4a,b) with wide variation as opposed to the expected trend of increasing residue levels along land surface temperatures hypothesized by Ma et al. [23]. Furthermore, the results in Table 3 indicate that the concentration of organophosphate pesticide residues in both cabbage and lettuce was higher in areas with moderate (Ashaiman) to high (Korle Bu) land surface temperatures compared to areas with lower (Atomic) land surface temperatures.

The Shapiro–Wilk test showed a non-normal distribution of the data. In contrast, the Kruskal–Wallis rank sum test variance in the composite pesticide residue (mean_Concentration) by site and vegetable revealed an insignificant difference (Kruskal–Wallis chi-squared = 2.1189, df = 5, *p*-value = 0.8325). However, the PERMANOVA results (Table 3 and Table 4) revealed differences across sites and the composition of the response variable. The significance of the PERMANOVA results implies that the site (temperature gradient) has a meaningful effect on the mean concentration of pesticide residues.

As shown in Table 3, the overall model’s F-statistic (1.9396, *p*-value 0.001) was statistically significant, meaning that site and pesticide type significantly influence the concentration of pesticide residues. The R² value of 0.0903 implies that site and pesticide type account for about 9.03% of the variation in the mean concentration of the pesticide residues.

In pairwise comparisons by site, none of the comparisons among the sites reveal significant differences (*p* > 0.05 in all cases). This indicates that the variation in the pesticide concentration is not predominantly influenced by the site alone; other factors, such as pesticide type, may exert a stronger effect. Several pairwise comparisons based on pesticide type show significant differences (*p* < 0.05), indicating that pesticide type significantly influences the data. Significant differences (*p* < 0.001) are found in comparisons of ACT vs. TMX < ACT vs. CHL < ACT vs. ACP < ACT vs. CHF < ACT vs. DMT < ACT vs. PRC, suggesting strong dissimilarities between these pesticide treatments.

Table 4 shows the results of the PERMANOVA test regarding differences in the mean concentration across vegetables and the composition of multiple pesticide residue (PR) variables. The R² value (0.0812) indicates that 8.12% of the variation in the model of the mean PR is explained by either the two vegetable types or the composition of the PR. The F-value (2.6751) indicates a strong model effect, with a highly significant *p*-value (0.001), confirming that the type of vegetable and composition of pesticide residues significantly affect the mean PR. The significant pesticide comparisons (*p* < 0.001) were ACT vs. CTD < ACT vs. TMX < ACT vs. CHL < ACT vs. ACP < ACT vs. CHF < CTD vs. TMX < CTD vs. CHL < CBZ vs. PRC < ACT vs. DMT < ACT vs. PRC < IMI vs. PRC < IMI vs. CHL. These comparisons suggest a strong compositional shift between these pesticides. However, there was no significant difference (*p* = 0.505) between cabbage and lettuce, meaning their impact on the mean PR concentration was similar.

### 3.2. Assessment of Public Health Risks of Pesticide Residues

**Figure 5:**
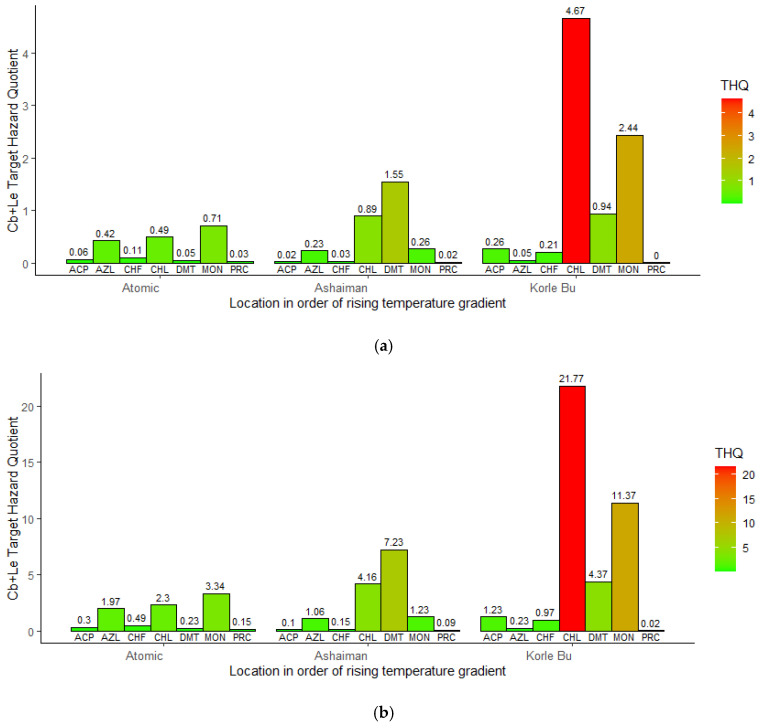
(**a**) Estimated adult target hazard quotient (THQ) of organophosphates in cabbage and lettuce (Cb + Le) by location. (**b**) Estimated child target hazard quotient (THQ) of organophosphates in cabbage and lettuce (Cb + Le) by location.

An assessment of the public health risks of pesticide residues revealed that a few pose a potential health risk to consumers (adults and children). The toxicity hazard quotient (THQ) revealed rising risks for adults (Figure 5a) and children (Figure 5b) exposed to organophosphates along rising land surface temperatures. The risk of exposure was higher for children than for adults. The estimated THQ for children (Figure 5b) in the areas with moderate (MON = 1.23; CHL = 4.16; DMT = 7.23) and high (ACP = 1.23; DMT= 4.37) temperature was more than one but less than ten, indicating a moderate risk of adverse health effects. At the high-temperature zone, the risk of exposure to MON (11.44) and CHL (21.77) was greater than 10, meaning there is a high risk of adverse health effects for children (Figure 5b). In contrast, adults (Figure 5a) showed a moderate risk of adverse health effects of exposure to MON (2.44) and CHL (4.67).

**Figure 6:**
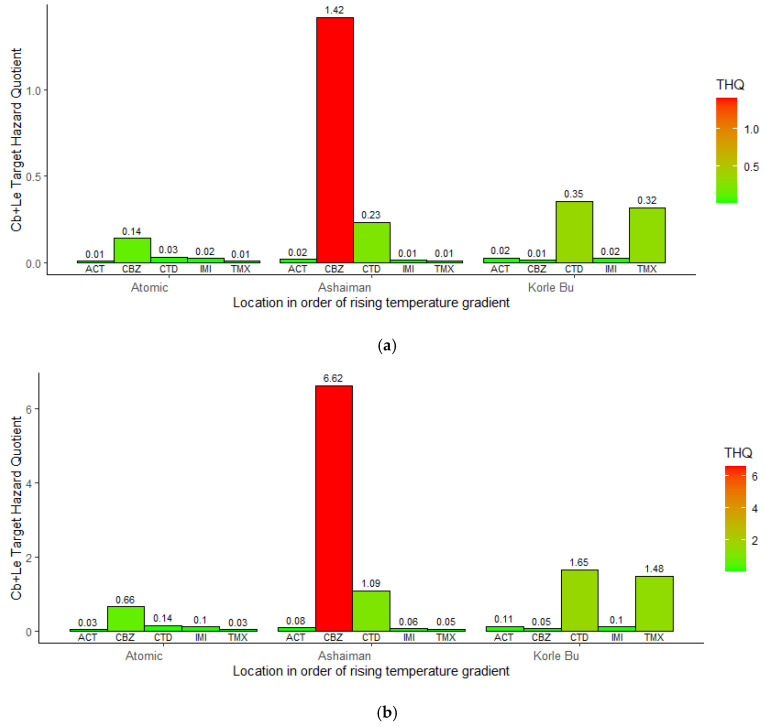
(**a**) Estimated adult target hazard quotient (THQ) of neonicotinoid/carbendazim in cabbage and lettuce (Cb + Le) by location. (**b**) Estimated child target hazard quotient (THQ) of neonicotinoid/carbendazim in cabbage and lettuce (Cb + Le) by location.

The estimated THQ for neonicotinoids/carbendazim pesticide residues in vegetables for adults (Figure 6a) and children (Figure 6b) was higher at moderate and high temperatures compared to the low temperature gradient. However, the risk of exposure was moderate (THQ (1 < THQ ≤ 10) for both adults (CBZ and CTD) and children (CBZ and CTD, TMX). The same applies to the child THQ at moderate (CTD = 1.09 and CBZ = 6.62) and high (CTD = 1.65 and TMX = 1.48) land surface temperatures. These results suggest a higher risk of adverse effects with increasing or high land surface temperatures. However, CBZ posed the most significant risk to adults and children at the moderate temperature gradient.

**Figure 7:**
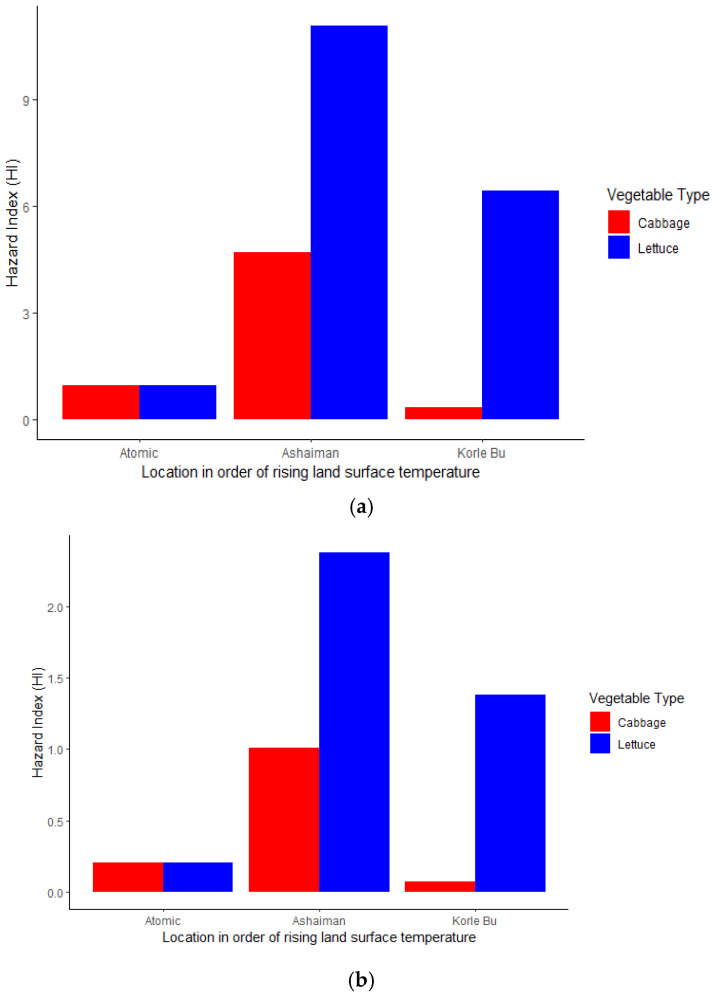
(**a**) Estimated neonicotinoid/carbendazim hazard index for children by location and vegetable. (**b**) Estimated neonicotinoid/carbendazim hazard index for adults by location and vegetable.

Generally, Figure 7 shows an increasing organophosphate hazard index for children (Figure 7a) and adults (Figure 7b) as temperatures and vegetable consumption increase. However, the cabbage trend exhibits an upward and downward HI trend. This implies that the risk is higher for lettuce than for cabbage along rising land surface temperatures.

**Figure 8:**
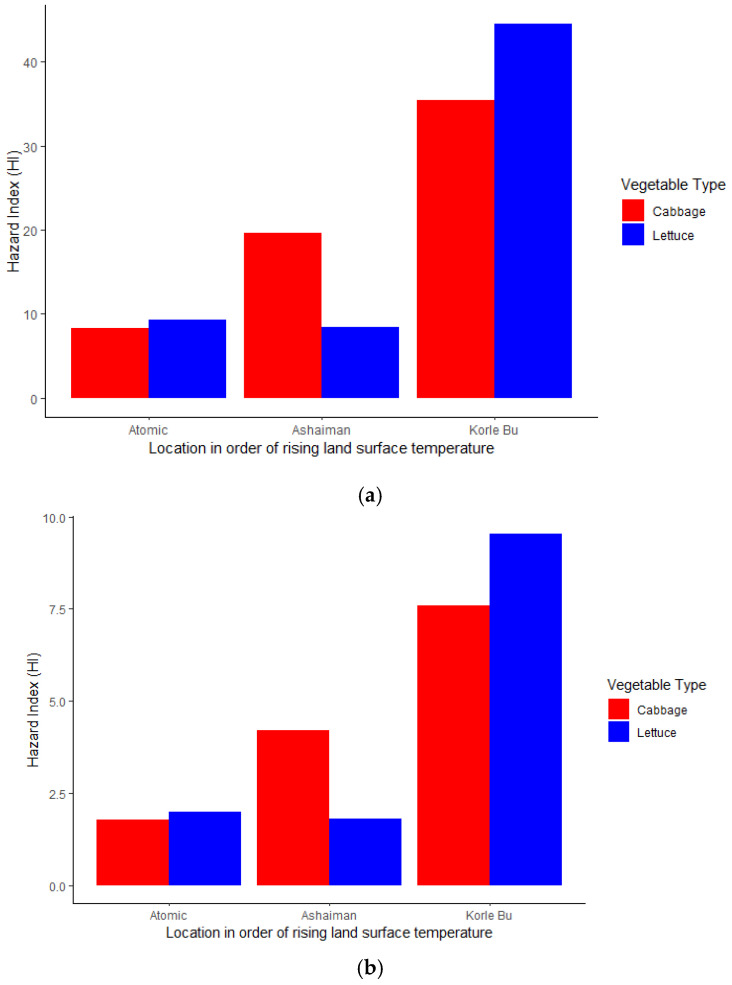
(**a**) Estimated organophosphate hazard index for children by location and vegetable. (**b**) Estimated organophosphate hazard index for adults by location and vegetable.

**Figure 9:**
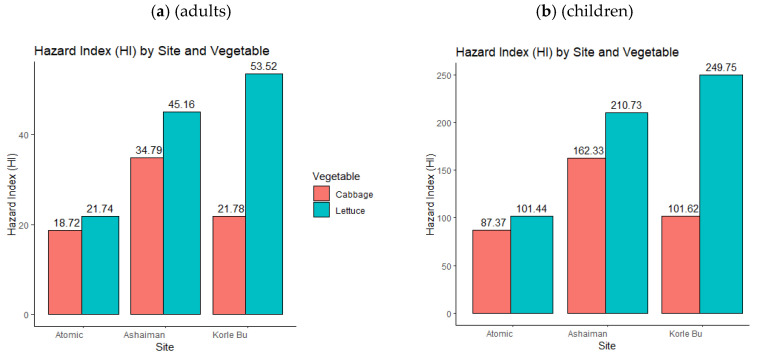
Combined organophosphate and neonicotinoid/carbendazim hazard index for (**a**) adults and (**b**) children across rising urban land surface temperatures.

Figure 8 shows an organophosphate hazard index for both children (Figure 8a) and adults (Figure 8b) across a temperature gradient and vegetables (cabbage and lettuce). The high temperature gradient (Korle Bu farm Site) had the highest risk of adverse health effects (HI > 10). In contrast, the moderate to low land surface temperatures fell within a moderate (1 < HI ≤ 10) hazard index. Essentially, these results imply that the higher the temperature gradient (Korle Bu farm Site), the higher the risk of adverse health effects of organophosphate exposure through cabbage and lettuce for adults and children in the study area (GAMA). The same trend was observed for the combined (aggregate) organophosphate and neonicotinoid/carbendazim hazard index for (a) adults and (b) children (Figure 9).

The overall hazard index (HI) for both adults and children in all the vegetable samples significantly exceeded the safety threshold (HI > 1), indicating potential severe health risks for individuals who consume these vegetables. Lettuce posed the highest risk compared to cabbage, suggesting that different vegetables have varying capacities for pesticide accumulation. Generally, the HI varied across sampling sites, showing an increasing trend of bioaccumulation with rising temperatures, particularly in lettuce. Vegetable samples from Korle-Bu had the highest HI, followed by Ashaiman and Atomic. This suggests that Korle-Bu had the highest pesticide contamination, which may pose a greater health risk to consumers in that area.

## 4. Discussion

According to Ma et al. [23], climate warming will increase crop pests and cause the intensive use of pesticides, pesticide resistance, and possible bioaccumulation in food crops. The present study reveals that individual pesticide residues vary across land surface temperatures. However, the cumulative concentration of the pesticide residues estimated by the target hazard quotient (THQ) and hazard index (HI) showed that a high urban temperature influences the accumulation of pesticide residues in lettuce more than cabbage, with significant public health implications for children compared to adults. The cumulative concentration of pesticide residues tends to increase along land surface temperature, particularly in lettuce compared to cabbage. This could be attributed to increased crop pests in warmer land surface areas, resulting in the intensive use of pesticides, subsequent pesticide resistance, and hence higher accumulation [23]. Individual concentrations of specific pesticide residues, such as CTD, IMI, ACP, TMX, CHF, and CBZ, exceed the WHO/EU’s maximum residue limits (MRLs), posing the highest human health risks to children compared to adults.

The Permanova analysis confirmed that while the land surface temperature of farm sites alone did not significantly influence the mean pesticide residue concentration, the composition of pesticide residues played a critical role in determining the overall contamination levels. The target hazard quotient (THQ) and hazard index (HI) assessments further revealed that children are at a higher risk of adverse health effects than adults, particularly in the high-temperature zone, where the composite concentration was the highest. This finding aligns with other research conducted by Gad Alla et al. [54] and Lozowicka et al. [55], indicating that children are more vulnerable to pesticide exposure. Although the high HI values estimated in the current study contradict those of Sharma et al. [22], the values affirm their assertion that children are more at risk than adults. The current findings reiterate the evidence presented by Lozowicka et al. [55] and Gad Alla et al. [54] concerning the long and short-term risks of excess pesticide residues in vegetables, which pose potential health risks to children and adults. The common pesticides detected by Lozowicka et al. [55] include organophosphates, methamidophos, carbamates, and methomyl. These reports and the findings of the current study demonstrate a need for stricter pesticide regulations and effective control programs to reduce pesticide residues to protect public health, primarily vulnerable populations like children in high-temperature zones.

In addition, the current findings align with the reports by Bloomfield et al. [24], Ma et al. [23], and Xi et al. [9], which indicate that pesticide residue accumulates in vegetables cultivated in warmer climates. Furthermore, the current study confirms the reports of Bloomfield et al. [24] and Ma et al. [23] showing that warmer temperatures delay the biodegradation of pesticide residues and facilitate bioaccumulation in tropical vegetables, often exceeding MRLs. The exceedance of the MRLs of CBZ, CTD, and TMX in moderate to high land surface temperature zones corroborates Quansah et al.’s [14] and Bempah et al.’s [2] reports of high levels of pesticide residues in Ghanaian vegetables, attributing this to excessive pesticide use, the use of groundwater, and inadequate regulation. The findings of the current study also support the findings of Struciński et al. [56], who demonstrated that organophosphates and carbamates are the most frequently detected pesticide residues in tropical vegetables in Poland, often exceeding regulatory thresholds. Additionally, the significant health risks associated with high pesticide concentrations in high land surface temperatures corroborate the research by Rhodes et al. [57], which showed that elevated temperatures exacerbate pesticide persistence and crop accumulation.

The current study aligns with other studies emphasizing the long-term health risks posed by vegetable pesticide residues. It confirms the findings of Shalaby et al. [58], who found that 63.1% of vegetable samples in Dakahlia, Egypt, contained pesticide residues, with HI (>1) values reaching up to 64% of the acceptable daily intake, highlighting the need for strict regulation [58]. Similarly, the study corroborates Yu et al.’s [59] reports that 23.4% of vegetable samples in Changchun, China, exceeded MRLs, with high contamination in leafy vegetables such as green onions and radishes. The current study also conforms to Lozowicka et al. [55], who reported that pesticide residues in vegetables posed a greater risk to children, with diazinon in lettuce and dieldrin in carrots contributing significantly to high HI values [55].

Beyond tropical settings, the present results affirm other studies in temperate regions, such as those by Leal Filho et al. [60] and Ulpiani [61], who demonstrated that urban heat islands can intensify the accumulation of contaminants. However, the HI values in temperate climates tend to be lower than those observed in tropical environments. These comparative insights highlight the unique vulnerability of urban tropical agriculture to pesticide-related health risks.

Although the present study did not compute carcinogenic health risks, several pesticides analyzed, including carbendazim (CBZ), Dimethoate (DMT), Chlorpyrifos (CHL), Prochloraz (PRC), Thiamethoxam (TMX), Acephate (ACP), and Chlorfenvinphos (CHF), have been classified as potential carcinogens by various regulatory authorities. According to Beyuo et al. [5], carbendazim (CBZ) is a well-known fungicide that causes liver and thyroid tumors

Carbendazim (CBZ), a widely used fungicide, has been identified as a probable carcinogen by the European Chemicals Agency (ECHA) due to its ability to interact with DNA and induce liver damage [48,62]. Similarly, Dimethoate (DMT), an organophosphate insecticide, has been linked to oxidative stress and an increased risk of non-Hodgkin’s lymphoma (NHL), leading the U.S. Environmental Protection Agency (EPA) to classify it as a likely carcinogen [63].

Chlorpyrifos (CHL), another organophosphate, has been associated with DNA damage and an elevated risk of childhood leukemia, leading it to receive a suggestive carcinogen classification according to the USEPA [62,64]. Prochloraz (PRC), a fungicide known for its endocrine-disrupting properties, has been recognized by the European Union (EU) as a potential carcinogen, particularly due to its metabolic disruption in animal studies [65].

Thiamethoxam (TMX), a neonicotinoid insecticide, has shown evidence of liver tumor formation in mice, leading to its classification as a likely carcinogen by the EPA [63]. Similarly, Acephate (ACP), an organophosphate insecticide, has been categorized as a possible carcinogen due to observed DNA damage, though available studies remain limited. Chlorfenvinphos (CHF), another organophosphate, has also been classified as a probable carcinogen by the US EPA, although its risk is somewhat mitigated by its restricted use and regulatory bans in several regions.

These findings highlight the potential human health risks associated with exposure to these pesticides, whether through food, water, or air. Given their widespread use, further research and regulatory scrutiny are essential to better understand their long-term health effects and to mitigate associated risks.

## 5. Conclusions

This study provides evidence that elevated urban temperatures in the Greater Accra Metropolitan Area (GAMA) are associated with higher concentrations of pesticide residues (PR) in cabbage and lettuce, which may pose increased public health risks, particularly for children. By analyzing both individual and multivariate pesticide concentrations alongside target hazard quotients (THQs) and hazard indices (HIs), the study offers important insights into the relationship between temperature variations and pesticide residue levels in commonly produced and consumed vegetables in cities.

This finding indicates that pesticide composition has significant influences on residue levels and spatial patterns, with statistically significant differences observed across farm sites with varying land surface temperatures. The results identify clear spatial trends in residue accumulation due to rising urban temperatures and associated health risks. Thus, we accept the research hypothesis that higher urban surface temperatures in the GAMA will lead to increased pesticide residues in cabbage and lettuce, posing public health risks to consumers. The most significant mean differences in pesticide residue concentration (*p* < 0.05) were observed in the following order: ACT vs. TMX < ACT vs. CHL < ACT vs. ACP < ACT vs. CHF < ACT vs. DMT < ACT vs. PRC. This indicates strong dissimilarities in pesticide accumulation across different sites. Additionally, significant compositional differences were found among specific pesticide combinations, including ACT vs. CTD, ACT vs. TMX, ACT vs. CHL, ACT vs. ACP, ACT vs. CHF, CTD vs. TMX, CTD vs. CHL, CBZ vs. PRC, ACT vs. DMT, ACT vs. PRC, and IMI vs. PRC. These findings highlight the nuanced compositional effects of pesticide residues and their interactions in agricultural environments.

Overall, the combined effects of individual pesticide residues had a critical influence on human health risk indices (THQ and HI), with children facing greater adverse health effects compared to adults. The evidence of increased pesticide residues in high-temperature zones underscores the urgent need for regulatory intervention. Authorities such as the Ministry of Food and Agriculture (MoFA), the Food and Drug Authority, and the Environmental Protection Agency should enforce stricter pesticide usage guidelines, particularly in urban farming areas, to mitigate health risks. Farmers should be encouraged to adopt Integrated Crop Pest Management (ICPM) strategies to reduce dependency on chemical pesticides, especially in warmer climates. Additionally, regular monitoring programs should be implemented to track pesticide residue levels and ensure compliance with safety standards. Public awareness campaigns should also be promoted to educate consumers on safe vegetable consumption practices, including thorough washing and the consideration of organic alternatives.

### Limitations and Future Research

While this study provides valuable insights, it is limited by the focus on specific vegetables and geographical areas (spatial extent). Future research should explore a broader range of spatial extents and vegetables through longitudinal studies to assess the long-term effects of pesticide exposure in varying climatic conditions [24]. Additionally, investigating alternative farming practices and mitigation strategies will be essential for reducing pesticide-related health risks associated with high temperatures in urban agricultural settings.

## Figures and Tables

**Table 1:** Distribution of vegetable farmers across study sites.

Study Site	Reference LST *	Classified	Verified Air Temperature	Cabbage Sample/Farmer	Lettuce Sample/Farmer	Total
Korle Bu		High	29.40 °C	11	11	22
Ashaiman		Moderate	28.17 °C	11	11	22
Atomic (Haatso)		Low	27.00 °C	11	11	22
Total				33	33	66

Note: LST * = land surface temperature; source: Gyimah et al. [10].

**Table 2:** Descriptions of pesticide residues and their human health effects.

Pesticide Active Ingredient	Description, Human Health Risk	Health Hazard	MRL (mg/kg)	RfD (mg/kg/day)	Agency
Acetochlor (ACT)	A chloroacetanilide herbicide for controlling grasses and broadleaf weeds, classified as a possible human carcinogen.	C	0.4	0.0039	[41,42]
Chlorothalonil (CTD)	A broad-spectrum fungicide used to protect vegetables from fungal diseases. Metabolite toxicity leads to strict residue limits in food.	NC	0.02	0.015	[41,43,44]
Thiamethoxam (TMX)	Neonicotinoid insecticide affects the nervous systems of insects. Outdoor use is banned by the European Union (EU) due to risks to pollinators.	C	0.02	0.007	[41,43,45]
Imidacloprid (IMI)	A neonicotinoid linked to pollinator decline and neurotoxic effects and reproductive issues.	NC	0.01	0.06	[41,43,46]
Carbendazim (CBZ)	A benzimidazole fungicide controlling fungal pathogens in vegetables. It causes endocrine disruption and reproductive toxicity.	NC	0.1	0.012	[47,48]
Acephate (ACP)	An organophosphate insecticide inhibiting acetylcholinesterase, affecting the nervous system and causing neurotoxicity concerns.	C	10	0.02	[41,43]
Azoxystrobin (AZL)	A strobilurin fungicide widely used in vegetable farming. It is safe within MRLs, and long-term exposure studies are ongoing.	NC	0.02		[41,47]
Chlorfenvinphos (CHF)	An organophosphate insecticide with neurotoxic effects. It is banned by the EU due to health risks but still used under Codex MRLs.	NC	0.01	0.0025	[41,43,45]
Dimethoate (DMT)	A systemic organophosphate insecticide with high acute toxicity. The EFSA banned its use in food crops, citing developmental neurotoxicity risks.	C	0.01	0.006	[49]
Monocrotophos (MON)	A highly toxic organophosphate insecticide. It is classified as extremely hazardous due to neurotoxic effects.	NC	0.01		[43,50]
Prochloraz (PRC)	An imidazole fungicide used to control fungal diseases in vegetables. It was assessed for its endocrine-disrupting potential.	C	0.03	0.009	[41,43,47]
Chlordane (CHL)	A chlorinated hydrocarbon insecticide with high persistence. It is banned globally under the Stockholm Convention on Persistent Organic Pollutants (SCPOP) due to carcinogenic and bioaccumulation.	C	0.01	0.0005	[43,51]

C = carcinogenic; NC = non-carcinogenic.

**Table 3:** Permanova results and pairwise comparison by site and composition of response variables.

Factor	Df	Sum of Squares	R²	F-Value	*p*-Value
1. Pairwise comparisons of mean pesticide residue concentration by site					
Atomic vs. Korle Bu	1	0.064	0.0011	0.5362	0.608
Atomic vs. Ashaiman	1	0.063	0.0012	0.5541	0.569
Korle Bu vs. Ashaiman	1	0.039	0.0007	0.3293	0.776
2. Pairwise comparisons of mean pesticide residue concentration by composition					
**Comparison**	**Df**	**Sum of Squares**	**R²**	**F-Value**	***p*-Value**
ACT vs. CTD	1	0.1833	0.0097	1.1537	0.285
ACT vs. TMX	1	0.3781	0.0285	3.4668	0.03 *
ACT vs. ACP	1	0.5134	0.0342	4.1764	0.024 *
ACT vs. CHF	1	0.4571	0.0382	4.682	0.013 *
ACT vs. DMT	1	0.6501	0.0555	6.9285	0.001 ***
ACT vs. PRC	1	0.8016	0.0775	9.9119	0.001 ***
ACT vs. CHL	1	0.4223	0.0323	3.9373	0.028 *

Distance matrix = the mean concentration of different pesticide residues. The permanova was estimated using “bray” method with permutations set at 999; *** = *p*-value < 0.001, * = *p*-value < 0.05. Acetamiprid (ACP), Clothianidin (CTD), Thiamethoxam (TMX), Carbendazim (CBZ), Acephate (ACP), Chlorfenvinphos (CHF), Dimethoate (DMT), Prochloraz (PRC), and Chlorpyrifos (CHL).

**Table 4:** Permanova results and pairwise comparison by vegetable and composition of response variables.

Factor	Df	Sum of Squares	R²	F-Value	*p*-Value
1. Permanova results: mean pesticide residue concentration by vegetable and composition					
Model	23	6.857	0.08122	2.6751	0.001 ***
Residual	696	77.563	0.91878		
Total	719	84.419	1		
2. Pairwise comparisons of mean pesticide residue concentration by vegetables					
Cabbage vs. Lettuce	1	0.077	0.00092	0.6576	0.505
3. Pairwise comparisons of mean pesticide residue concentration by composition					
ACT vs. CTD	1	0.1833	0.0097	1.1537	0.297
ACT vs. TMX	1	0.3781	0.0285	3.4668	0.035 *
ACT vs. ACP	1	0.5134	0.0342	4.1764	0.018 *
ACT vs. CHF	1	0.4571	0.0382	4.682	0.01 **
ACT vs. DMT	1	0.6501	0.0555	6.9285	0.004 **
ACT vs. PRC	1	0.8016	0.0775	9.9119	0.002 **
ACT vs. CHL	1	0.4223	0.0323	3.9373	0.026 *
CTD vs. TMX	1	0.6956	0.0411	5.0672	0.008 **
CTD vs. CHL	1	0.7235	0.0430	5.340	0.004 **
IMI vs. PRC	1	2.1752	0.1423	19.578	0.001 ***
IMI vs. CHL	1	1.4291	0.0810	10.395	0.001 ***
CBZ vs. PRC	1	0.5363	0.0469	5.8016	0.007 **

Distance matrix = the mean concentration of different pesticide residues. The permanova was estimated using the “bray” method with permutations set at 999; *** = *p*-value < 0.001, ** = *p*-value < 0.01, * = *p*-value < 0.05. Acetamiprid (ACP), Clothianidin (CTD), Thiamethoxam (TMX), Acephate (ACP), Chlorfenvinphos (CHF), Dimethoate (DMT), Prochloraz (PRC), Chlorpyrifos (CHL), Imidacloprid (IMI), Carbendazim (CBZ).

## Data Availability

The original contributions presented in this study are included in the article. Further inquiries can be directed to the corresponding author.
